# Efficient removal of mercury and chromium from wastewater *via* biochar fabricated with steel slag: Performance and mechanisms

**DOI:** 10.3389/fbioe.2022.961907

**Published:** 2022-08-25

**Authors:** Huabin Wang, Ran Duan, Xinquan Zhou, Jia Wang, Ying Liu, Rui Xu, Zhuwei Liao

**Affiliations:** ^1^ School of Energy and Environment Science, Yunnan Normal University, Kunming, China; ^2^ Department of Environmental Engineering, School of Environmental Science and Engineering, Huazhong University of Science and Technology, Wuhan, China; ^3^ School of Chemical Engineer and Pharmacy, Henan University of Science and Technology, Luoyang, China; ^4^ Urban Construction Engineering Division, Wenhua College, Wuhan, China

**Keywords:** biochar, steel slag, mercury, chromium, heavy metals, adsorption, immobilization

## Abstract

Biochar derived from biomass is regarded as a promising adsorbent for wastewater treatment, but the high cost of modification is still a challenge for its large-scale practical applications. In this study, we employed steel slag as a low-cost fabricant and synthesized hydrothermally carbonized steel slag (HCSS), as a stable environmentally functional material for heavy metal removal. Typically, positively and negatively charged heavy metal contaminants of Hg^2+^ and Cr_2_O_7_^2−^ were employed to testify the performance of HCSS as an adsorbent, and good capacities [(283.24 mg/g for Hg (II) and 323.16 mg/g for Cr (VI)] were found. The feasibility of HCSS on real wastewater purification was also evaluated, as the removal efficiency was 94.11% and 88.65% for Hg (II) and Cr (VI), respectively. Mechanism studies revealed that the modification of steel slag on bio-adsorbents offered copious active sites for pollutants. As expected, oxygen-containing functional groups in HCSS acted as the main contributor to adsorption capacity. Moreover, some reactive iron species (i.e., Fe^2+^) played an essential role in chemical reduction of Cr (VI). The adsorptive reactions were pH-dependent, owing to other more mechanisms, such as coprecipitation, ion-exchange, and electrostatic attraction. This promising recycling approach of biomass waste and the design of agro-industrial byproducts can be highly suggestive of the issues of resource recovery in the application of solid waste-derived environmentally functional materials for heavy metal remediation.

## 1 Introduction

Heavy metal pollution in the aqueous system brought detrimental effects to human beings as well as aquatic lives, which has attracted much attention in recent years. Many approaches were adapted for these water contaminants elimination (Zhang et al., 2018; Zhu et al., 2019), and among these methods, adsorption was regarded as an economic-effective, simple operated, and less-second-pollution one (Lin et al., 2019; Xu et al., 2019; Yang et al., 2019). Hydrothermal carbonization has emerged as a promising method for bio-adsorbents preparation, as this process could simultaneously achieve the targets of resource recovery and environmental protection (Huang et al., 2017). The exceptional adsorptive properties of hydrochar were owing to the copious oxygen-containing functional groups, such as hydroxyl, phenolic, and carbonyl (Xia et al., 2019). However, these carbonaceous fractions were lack of stability in the aqueous system, causing them to partially dissolve into the solution. To solve this problem, some posttreatment was conducted to stabilize them, such as pyrolysis or calcination (Johs et al., 2019).

In addition to heat treatment, various raw materials were applied as carbon support to synthesize hybrid composites with hydrochar components, such as polymers (Ghadikolaei et al., 2019), layered double hydroxides (Zhang et al., 2018), etc., Another category of supporting materials was inorganic metal oxides, the regular structure and abundant surface hydroxyl groups provided sufficient active sites for organic components adherence. Some metal oxides, including manganese oxide (Li et al., 2019) and ferromanganese oxide (Zhou et al., 2018), were employed for the synthesis of metal-organic complexations. Moreover, some solid waste containing metal oxides were also applied as carbon support. Our group attempted to reutilize the sewage sludge, as a carbon support, to capture heavy metals from wastewater (Ngambia et al., 2019), which provided a novel strategy for support design and solid waste reutilization.

Other promising support, such as steel slag, which was often recognized as a solid waste generating from metallurgy, also might be a carbon support as its relatively inert physicochemical property (Gómez-Nubla et al., 2018). Nowadays, the most common treatment of steel slag was landfilling (more than 70 wt.%), which accumulated a large number of metallic elements deposited in fields, leading to the contamination of soil and correspondingly potential risks to public health (Qasrawi, 2018). Many approaches were adapted to reutilize steel slag and its derivatives. Fang et al. (2018) converted slag into porous calcium silica hydrate *via* a simple hydrothermal process and then conducted these slag-derived materials for phosphate immobilization from wastewater. These functional materials demonstrated distinct skeleton structures and abundant active sites which contributed to the pollutants adsorption. Meanwhile, Czech et al. (2018) implemented heat treatment to fabricate calcium silicate-rich slags and then these materials were conducted for cadmium removal from wastewater. Moreover, steel slag was also employed as a support for sodium hydroxide, to enhance the precipitation and coagulation of phosphorus (Park et al., 2017). The sodium hydroxide coated on the surface of steel slag maintained the high pH (>8) of surroundings and subsequently enhanced the removal performance.

Furthermore, hydrothermal treatment was proved as an effective approach to lattice perfection and accelerating crystallization (Zhang et al., 2019). Liu’s group applied the hydrothermal method to treat wasted gypsum, and they found this solid waste could be converted and recrystallized, implying hydrothermal treatment could accelerate crystallinity of solid waste (Liu et al., 2018). In addition to structure reconstruction, some chemically active species in steel slag may be also activated after co-hydrothermal process and then reacted with pollutants. Nelson’s group proved Fe (II) species in ores or silica surfaces could react with Cr (VI) in solution, as an electron donor for the chemical reduction of Cr (VI) (Nelson et al., 2019). Wang’s group also found natural polyphenols system containing Fe (III) could react with Cr (VI), and the *in situ* generated Fe (II) in aqueous media played a vital role for reduction process (Hu et al., 2019b). Hence, in our study, sawdust, as an ordinary biomaterial was applied to represent the biomass feedstock, could be converted into the hydrochar components under the hydrothermal circumstance. Meanwhile, stable inorganic skeleton derived from steel slag might provide the copious active sites for organic components adherence, leading to the large density of surface functional groups, which might play a crucial role in chemisorption and improve the removal performance.

In this study, a novel hydrothermally carbonized steel slag (HCSS) was synthesized *via* a facile one-pot method and conducted for Hg^2+^ and Cr_2_O_7_^2−^ removal, which was typical positively and negatively charged contaminant, respectively. Batch adsorption experiments were conducted to evaluate the environmental performance of this adsorbent as a function of reaction dosages, contact time, and regeneration test, and this feasibly prepared sorbent exhibited a superior capacity for Hg^2+^ and Cr_2_O_7_^2−^ removal. In addition, the industrial applicability of HCSS on practical wastewater purification was also evaluated. Elemental analysis, XRD, XRF, SEM, and other approaches were employed and revealed the multiple reaction mechanisms.

## 2 Materials and methods

### 2.1 Materials

NaOH, HNO_3_ (68.0%), HCl (36.0%–38.0%), NaHCO_3_, NaCO_3_, CaCl_2_, Cd(NO_3_)_2_, K_2_Cr_2_O_7_, HgCl_2_, MgSO_4_, and NaCl were obtained from Sinopharm Chemical Reagent Co., Ltd. All reagents used in batch experiments were of analytical reagent grade and used as-received without any further purification. Steel slag (SS) samples were collected from Wuhan Iron and Steel (Group) Company, categorized as basic oxygen slag. SS samples were washed and dried at 50°C overnight and then these slags were smashed and passed through a 0.45-mm sieve. According to relevant literature, steel slag is an alkaline material; the chemical composition of steel slag was determined by XRF. The chemical composition (wt%) of SS is shown in Table 1. Sawdust was collected from a furniture company, located in Wuhan city, and this obtained pinewood sawdust was smashed and crushed through a 0.15-mm sieve. Practical Hg (II)-containing wastewater was collected from a local foundry industry, while polluted water containing Cr (VI) was gathered from an electronic industry in Wuhan city. Deionized water was applied through all the batch experiments, as 18.0 mΩ cm, generated by using a Milli-Q system.

**TABLE 1:** Chemical composition of steel slag used in this study.

Element	Wt%	Element	Wt%
Ca	25.49	As	0.10
Si	44.78	Cd	0.25
Al	18.76	Pb	0.26
Mg	3.52	C	0.23
Fe	7.91	S	0.45
Cr	0.21	N	0.15
Ni	0.15		

### 2.2 Characterization

The composition of adsorbents was analyzed by using the X-ray fluorescence method (XRF, EAGLE III, EDAX Inc., United States) and inductively coupled plasma-optical emission spectrometry (ICP-OES, Optima 8,300, PerkinElmer, United States), after digestion by the HNO_3_/H_2_O_2_/HClO_4_ (2:2:1) mixed solution for 24 h. The compositions of C, N, and S were measured using an elemental analyzer (EA, Vario MICRO cube Elementary, German). The X-ray diffraction spectra were conducted by X’Pert PRO (PANalytical B.V, Holland), with Cu Kα radiation (λ = 0.1542 nm) over a 2ϴ collection range of 5–80 with a scanning rate of 5 /min. The surface area and pore size measurements were conducted by a JW-BK low-temperature N_2_ adsorption instrument (JWGB Sci. &Tech, China) at −196.15°C, and the Brunauer–Emmett–Teller equation was used to calculate the surface area. Before measurement, the sample was degassed under vacuum and dried at 120°C for 24 h. The pH was adjusted by adding 0.01 mM NaOH or 0.01 mM HCl into the solution. The FT-IR spectrum was detected by using the KBr pellet method, over the wavelength ranging from 4,000 to 400 cm^−1^ by using a Vertex 70 Fourier transform infrared spectrometer (United States). Quantitative calculation of functional groups was characterized by Boehm titration. In brief, 1.0 g HCSS was added into a 100 ml Erlenmeyer flask containing 25 ml NaOH (0.05 M), shaken for 24 h, filtrated, and then washed with deionized water for three times; the washed water was collected for analysis. For integrity, deionized water without samples was used as blanks. In addition, 0.1 g methyl red was dissolved into 100 ml ethanol as an indicator and added into solution with 0.15 ml for each sample. Furthermore, 0.05 M HCl was used for titration, consumed amount of HCl solution at the equilibrium state was used for calculation, and then the total amount of acidic groups on the surface of adsorbents was calculated. Moreover, NaHCO_3_ (0.05 M) and Na_2_CO_3_ (0.05 M) were conducted to test and calculate the amount of carboxyl and hydroxyl with the same former process. A scanning electron microscope (SEM, Quanta 200 ESEM FET, Holland) cooperated with dispersive electron X-ray (EDX) equipment was applied to elucidate the morphology of the adsorbent. X-ray fluorescence (XRF, EAGLE III, EDAX Inc., United States) was applied for characterization of HCSS after reactions. The pH_PZC_ and zeta potentials of HCSS at various pHs were measured by Zetasizer Nano series (Nano-ZS90, Malvern, United Kingdom) under 25°C. First, 150 mg of HCSS was individually added into plastic conical flasks containing 65 ml deionized water. Second, the mixture was mechanically shaken for 24 h. Then the resultant materials were collected for zeta potential determination by applying 0.01 mM NaCl solution as the background.

### 2.3 Preparation

#### 2.3.1 HCSS

**SCHEME 1:**
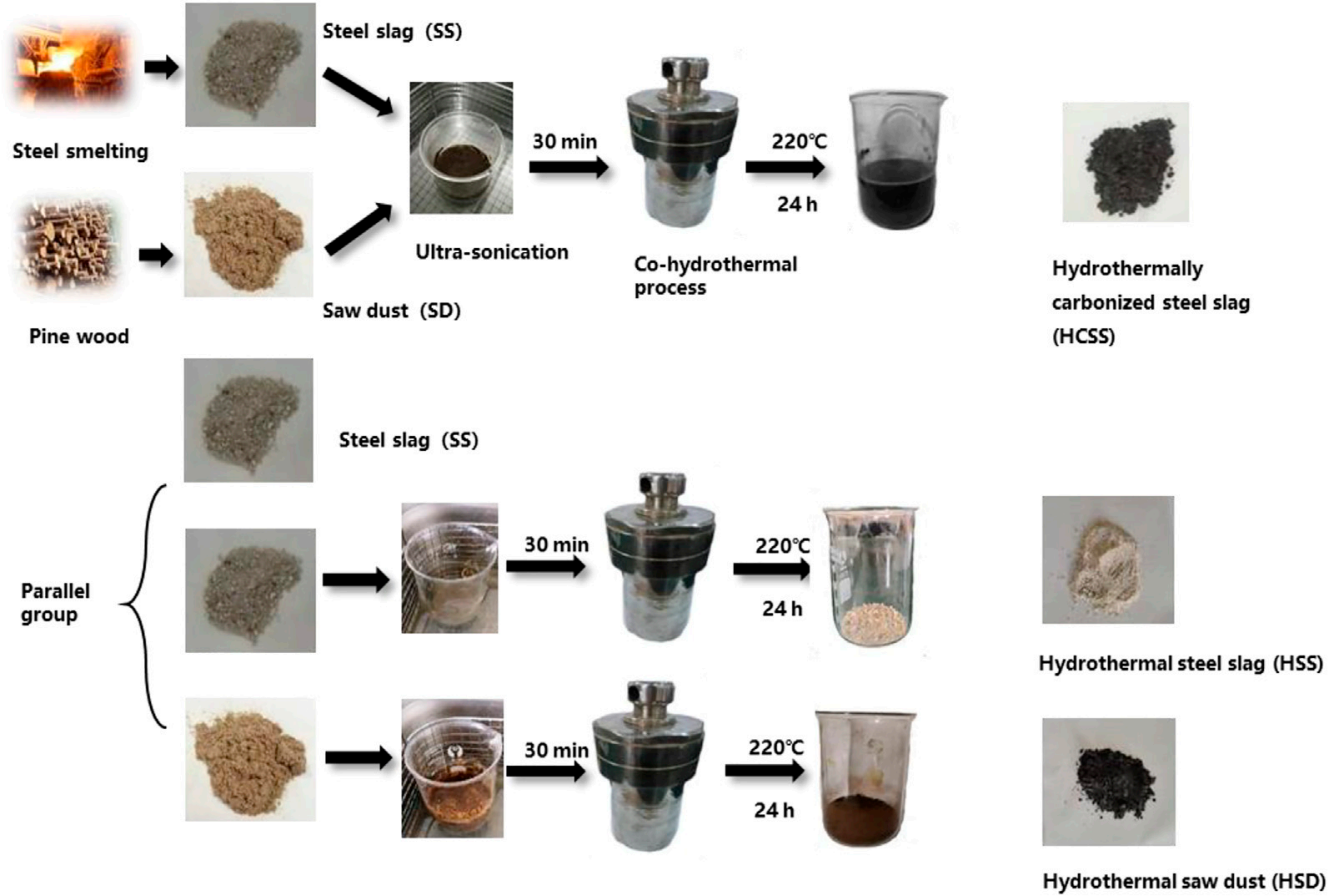
Preparation of HCSS and the parallel samples.

General preparation methods of hydrothermally carbonized steel slag (HCSS) and parallel materials are shown in Scheme 1. Specifically, 3.75 g pinewood sawdust (SD) and 3.75 g steel slag (SS) were soaked in 56 ml aqueous solution (weight ratio of SD:SS = 1:1). A volume of 19 ml NaOH solution (1 M) was slowly added, forming a solution with 10% solid part in weight, and was kept stirring at room temperature (25°C) at 200 rpm for 30 min. Then this mixture was transferred into a 250-ml stainless steel autoclave for the hydrothermal process at 220°C for 24 h. After hydrothermal treatment, the mixture was filtrated, washed with deionized water several times, dried in a vacuum oven at 80°C for 6 h, and stored for further experiments. Additionally, different ratios of SD and steel slag SS were conducted to manipulate the properties of adsorbents. The weight ratio of SD:SS varied from 1:2, 1:1, and 2:1, which were labeled as HCSS2, HCSS, and HCSS0.5, respectively. Similarly, different heating temperatures were also conducted with the ratio of 1:1, and HCSS300, HCSS, and HCSS500 were prepared under 300°C, 400°C, and 500°C, respectively.

#### 2.3.2 Parallel group

As shown in Scheme 1, other materials were parallelly prepared for comparison. All conditions were identical to the preparation of HCSS. Hydrothermal steel slag (HSS) was prepared with SS without the addition of sawdust, while hydrothermal sawdust (HSD) was prepared with only sawdust but no SS.

### 2.4 Batch experiments

#### 2.4.1 Adsorption behaviors

The adsorptive capacity of Hg (II) and Cr (VI) by HCSS was determined by metal capacity values (mg/g). The effects of dosages on adsorption performance of Hg (II) and Cr (VI) from the aqueous system on selected adsorbents were investigated. A volume of 2–20 mg of HCSS was added into 10 ml Hg (II) solution (100 ppm) or 10 ml Cr (VI) solution (100 ppm) in vials at room temperature (25°C) for 24 h. The influence of pH (2.0–9.0) tests was conducted by adding 10 mg HCSS to 10 ml of Hg (II) and Cr (VI) solutions (100 ppm) at 25°C, and neglectable volumes of 0.1 M NaOH or HNO_3_ was added for adjusting pH to desired values. A kinetics study was performed by adding 10 mg HCSS into 10 ml Hg (II) and Cr (VI) solution (400 ppm) at 25°C for different time intervals (10, 20, and 30 min and 1, 6, 8, 12, 24 h). Correspondingly, isotherm experiment was carried out by mixing 10 mg HCSS with 10 ml solution in the range of 10–1,000 ppm Hg (II) and Cr (VI) at 25°C. Evaluation of leaching performance was also conducted; SS, HSS, and HCSS were leached at a liquid to solid (L/S) ratio of 10 L/kg for 48 h in acid-cleaned 100 ml PRFE vessels, continuously stirred, and kept at a constant temperature of 25°C. The release of the mercury- and chromium-adsorbed HCSS was conducted by adding 0.1 M Na_2_EDTA solution as eluent at 25°C with HCSS dose of 2 g/L. The adsorbed HCSS was stirred in Na_2_EDTA for 1 h, washed by deionized water three times, and then dried in a vacuum oven at 105°C for next recycle. In order to evaluate the stability of HCSS, residual weight after six cycles was also tested. Once the reaction was experimented thoroughly, the mixtures were filtered through a 0.22-μm pore size nylon filter and the mercury and chromium concentration in samples was detected by inductively coupled plasma-optical emission spectrometry.

The adsorptive capacity (q_e_) for Hg (II) and Cr (VI) after the reaction is calculated according to the following equation:

$$\begin{array}{c} q_{e}=\frac{\left(C_{0}-C_{e}\right)V}{m} \end{array},$$ (1)

where C_0_ and C_e_ are the concentrations of Hg (II) (ppm) and Cr (VI) (ppm) for initial and equilibrium conditions, respectively; V is the volume of solution (L); and m is the mass of adsorbents (g).

#### 2.4.2 Modeling

The kinetics of Hg(II) and Cr(VI) removal was assessed using two models, the pseudo-first-order model and pseudo-second-order model, which were conducted for analyzing the mechanisms of the reaction.

The pseudo-first-order model is as follows:

$$\begin{array}{c} ln\left(q_{e}-q_{t}\right)=lnq_{e}-k_{1}t \end{array}.$$ (2)

The pseudo-second-order model is as follows:

$$\begin{array}{c} t/q_{t}=1/k_{2}q_{e}^{2}+{\left(1/q_{e}\right)}^{t} \end{array},$$ (3)

where q_t_ and q_e_ is the amount of Hg (II) and Cr (VI) captured by HCSS (mg/g) at time t and equilibrium, respectively; the k_1_ (min^−1^) and k_2_ (g/mg min^−1^) are the constants for the pseudo-first-order and pseudo-second-order model, respectively.

The adsorption isotherm was appraised by two models: Langmuir model and Freundlich model.

The Langmuir model is as follows:

$$\begin{array}{c} C_{e}/Q_{e}=C_{e}/Q_{m}+1/Q_{m}K_{L} \end{array},$$ (4)

where Q_e_ is the capacity of HCSS on Hg (II) and Cr (VI) elimination (mg/g), C_e_ is the equilibrium concentration of target pollutants (mg/L), Q_m_ is the calculated maximum capacity of HCSS (mg/g), K_L_ is the Langmuir coefficient, and plots of C_e_/Q_e_ plotted versus C_e_ could be a straight line as the slope was 1/Q_m_ and the intercept was 1/Q_m_K_L_.-

The Freundlich model is as follows:

$$\begin{array}{c} logQ_{e}=logK_{F}+1/nlogC_{e} \end{array},$$ (5)

where K_F_ and n is the calculated capacity of HCSS by Freundlich (mg/g) and intensity of reactions, respectively; C_e_ is the initial concentration of Hg (II) and Cr (VI) in solution (mg/L).

## 3 Results and discussion

### 3.1 Characterization

Initial comparison of HSS, HSD, HCSS, and other slags was conducted by evaluating the porous properties, including S_BET_, average pore volume, or width, as listed in Table 2. Without the incorporation of bioresource of sawdust, S_BET_ values of blast furnace slag, acid- and alkali-treated slag, and HSS were observed extremely limited (4.26, 3.46, and 2.54 m^2^/g, respectively), which was due to its inorganic nature (Gao et al., 2017). On the other hand, HSD exhibited satisfying surface area of 101.46 m^2^/g due to the mesoporous structure generated by structure recrystallization. Interestingly, the SBET value of HCSS (52.84 m^2^/g) was even larger than the sum of HSS (2.54 m^2^/g) and HSD (43.29 m^2^/g). The significant increase of S_BET_ for HCSS might be explained by the organic components emerged during the transformation of biomass and then anchored onto the surface of slag skeleton, with the function of relatively high temperature and self-generated pressure (Zhu et al., 2019). Correspondingly, the pore volume of HSS (0.021 cm3/g) also increased to 0.055 cm^3^/g of HCSS with the incorporation of sawdust. However, the average pore width dropped from 330.07 Å (HSS) to 41.63 Å (HCSS), implying that HCSS possessed a larger surface area and average pore volume but smaller pore widths.

**TABLE 2:** Comparison of the number of surface parameters, functional groups, and residual weight of HSS, HSD, and HCSS with other adsorbents.

Sample	S_BET_ (m^2^/g)	Average	Average	Amount of functional groups (mmol/g)			Residual weight after recycles^c^ (%)	Reference
		pore volume^a^ (cm^3^/g)	pore width^b^ (Å)	Carboxyl	Lactone	Hydroxyl		
Blast								Gao et al. (2017)
Furnace slag (BFS)	4.26	0.005	47.00	—	—	—	—	
BFS								
Acid–alkali precipitate	3.46	0.013	154.00	—	—	—	—	
HSS	2.54	0.021	330.07	0.061	0.026	0.087	98.6	This work
HSD	43.29	0.113	101.46	0.340	0.205	0.675	44.3	
HCSS	52.84	0.055	41.63	0.439	0.374	0.322	96.2	

a Pore volume determined at P/P_0_ = 0.99.

b Adsorption average pore width (4V/A by BET).

c After recycled for six times. Conditions: DI; water volume: 10 ml; initial dosage: 1.0 g/L; reaction time: 24 h; temperature: 25°C, filtrated, and dried for the next cycle.

It was widely accepted that the surface functional groups played pivotal roles in the adsorption of heavy metals. Therefore, the quantitative study of functional groups was employed by Boehm titration, and the total number of carboxyl, lactone, and hydroxyl is summarized in Table 2. In terms of carboxyl, HCSS exhibited higher density compared with HSS or HSD (0.439, 0.061, and 0.340 mmol/g for HCSS, HSS, and HSD, respectively). The same trend was observed for lactone (0.374 mmol/g for HCSS, 0.096 mmol/g for HSS, and 0 0.005 mmol/g for HSD) and hydroxyl (0.322 mmol/g for HCSS, 0.087 mmol/g for HSS, and 0.675 mmol/g for HSD). The increasing of functional groups on HCSS also demonstrated the successful assembly of the hydrochar component onto the surface of slag skeleton (Yin et al., 2017).

Moreover, the residual weight of these adsorbents after recycling six times was also collected for evaluating the stability (as shown in Table 2). After recycling several times, 98.6% of weight remained for HSS, indicating the stable physicochemical property of this solid waste. Expectedly, after being recycled, the HSD only had 44.3% of the weight left suggesting that the organic component could be partially dissolved in the solution, resulting in the loss of HSD (Ahmad et al., 2019). However, after several recycles, the synthesized HCSS had 96.2% of weight remained, implying the excellent stability of the organic component could be substantially improved after being supported by SS.

### 3.2 Adsorption behavior

#### 3.2.1 Adsorbent comparison

**FIGURE 1:**
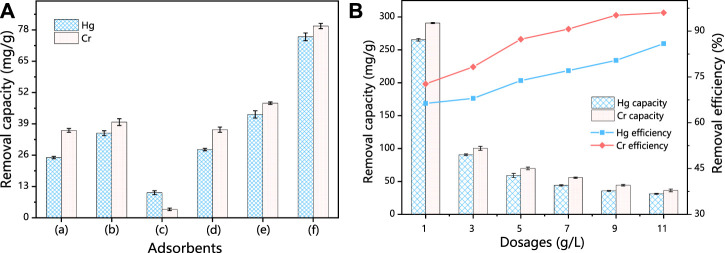
**(A)** Hg(II) and Cr(VI) removal in different systems. **(B)** Influence of different dosages for the capacities and efficiency. Conditions: C0 = 400 ppm; sample volume: 10 ml; reaction time = 24 h; pH 6.5; and temperature: 25°C.

Initially, the adsorption capacity for Hg (II) and Cr (VI) was compared between SS, HSS, HSD, HCSS, and physical mixture of HSS/HSD and SS/HSD as the parallel samples (Figure 1A). SS alone exhibited a capacity of 25.0 mg/g for Hg (II) and 36.7 mg/g for Cr (VI), and due to the certain porosity and specific surface area of SS, Hg (II) and Cr (VI) can be adsorbed into the steel slag skeleton by an intermolecular force. Meanwhile, the hydroxyl group (OH^−^) and other groups also could be involved with the adsorption. These sites can attract pollutants by electrostatic adsorption, so the original steel slag also has a certain adsorption capacity (Duan & Su, 2014; Wang et al., 2018). Also, after being converted from SS into HSS, the capacity increased to 35.1 and 39.9 mg/g for Hg (II) and Cr (VI), respectively. The better performance of HSS could be attributed to the incompletely reformed structure and functional groups on its surface, which complied with Boehm titration. In the case of HSD alone, the removal capacity was 11.4 and 4.4 mg/g for Hg (II) and Cr (VI), respectively, and these unsatisfied values might be assigned to the constrained active cites and low functional group density on the surface of hydrochar (Zhou et al., 2018). Moreover, in the case of HSD alone, after reacting for 24 h, the solution turned into dark brown and still light brown after filtration, indicating that the organic component was partially dissolved and reduced the practicality of HSD alone (Supplementary Figure S1). To further testify the function of biomass feedstock, the mixture of SS and HSD was employed, and the limited increase of capacity compared with SS alone was achieved [from 25.0 to 28.3 mg/g for Hg (II) and from 36.7 to 42.9 mg/g for Cr (VI)]; this could be contributed to the addition of organic functional groups. Same results for the system that mixed HSS with HSD were also obtained, and the capacity was increased from 35.1 to 42.9 mg/g for Hg (II) and from 39.9 to 47.7 mg/g for Cr (VI). However, in the case of HCSS alone, the superior capacity of 75.1 mg/g for Hg (II) and 82.7 mg/g for Cr (VI) was achieved, which was considerably improved compared with other sorbents. This synergetic effect could be explained by the multiple interactions between extra components that emerged during the transformation of co-hydrothermal treatment of both SD and SS in HCSS (Khan et al., 2019). According to the specific affinity between functional groups with surface metal ions, the introduced biomass feedstock could be converted into functional groups, and the regular crystalline structure was also beneficial to pollutants precipitation. On the other hand, the inorganic skeleton as carbon support and provided copious active sites for the functional group adherence increased the density of surface functional groups, which was consistent with aforementioned physicochemical characterizations.

In order to investigate the optimum mass ratio between SS and SD, various adsorbents were prepared in different mass ratios as HCSS2 (SD:SS = 1:2), HCSS (SD:SS = 1:1), and HCSS0.5 (SD:SS = 2:1). As shown in Supplementary Figure S2, the capacity increased from 66.98 mg/g and of HCSS2 to 75.12 mg/g of HCSS for Hg (II), indicating the increased amount of organic components could improve the adsorptive performance. After more SD was added during preparation, the SBET of HCSS was increased, and more functional groups were obtained. However, the capacity of HCSS0.5 (50.03 mg/g) was decreased compared to HCSS (75.12 mg/g), this could be attributed to that loading of too many organic ingredients might occupy the active sites on the surface of slag skeleton, decreasing of capacity for heavy metals removal. Same trend was observed for Cr (VI) removal, capacity increased from 49.12 mg/g (HCSS2) to 82.69 mg/g (for HCSS) and then decreased to 65.12 mg/g (for HCSS0.5). Therefore, the mass ratio as SD:SS = 1:1 (HCSS) was selected as the ideal value for adsorbent synthesis.

Furthermore, effects on adsorptive performance by heat treatment temperatures were also evaluated (Supplementary Figure S2B). Adsorbents synthesized with different temperatures were labeled as HCSS300, HCSS, and HCSS500, representing the preparing temperatures were 300°C, 400°C, and 500°C, respectively. In the case of mercury removal, at 300°C, the relatively low temperature made the introduced biomass raw materials not fully carbonized, the pores were not well developed, and they cannot totally react with mercury. The capacity increased from 63.10 mg/g of HCSS300 to 75.12 mg/g of HCSS, which might be attributed to the fact that high temperature was beneficial for increasing of the aromatic structure and S_BET_ values of adsorbents, thus increasing the contact between biochar and Hg^2+^ (Dai et al., 2019). Meanwhile, HCSS500 exhibited decreased capacity (46.81 mg/g) compared to HCSS300 or HCSS, demonstrating the high temperature was detrimental to the capacity. This phenomenon was ascribed to the loss of biomaterials by calcination under this condition, which led to the reduction of organic functional groups and the destruction of some pore structures, thus reducing the adsorption capacity of mercury. On the another hand, the Cr (VI) removal rates of HCSS300, HCSS, and HCSS500 also showed the same trend, and the capacities were 69.08 mg/g, 82.69 mg/g, and 80.58 mg/g for HCSS300, HCSS, and HCSS500, respectively. There was a neglectable capacity decrease on Cr (VI) removal (from 82.69 of HCSS to 80.58 mg/g of HCSS500) compared to the decreased values for Hg (II) removal, which suggested the limited influence of the organic components on the performance of Cr (VI) removal. It can be seen that the pyrolysis temperature determines the physicochemical properties of the adsorbent and has a significant effect on its adsorption capacity. Therefore, 400°C was selected as the ideal temperature for adsorbents synthesis and prepared HCSS was employed for further adsorption analysis.

#### 3.2.2 Dosage effects

As shown in Figure 1B, the elimination efficiency of Hg (II) was increased from 66% to 79% with the dosage increased from 1 to 9 g/L and then remained higher than 80% when the dosage reached 11 g/L. However, the maximum capacity of 265.3 mg/g was achieved, and the capacity decreased with the increase of dosage. As for Cr (VI) removal, with the increase of dosage of HCSS, the adsorptive capacity was decreased from 290.8 mg/g to 36.4 mg/g, while the removal efficiency increased from 75% to 95% with the dosage increasing from 1 to 11 g/L.

**FIGURE 2:**
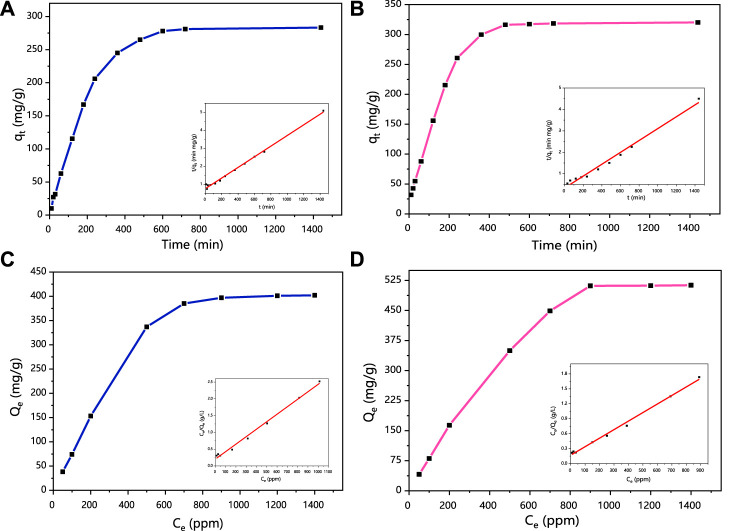
**(A)** Adsorption kinetics curves of HCSS for Hg (II) removal. **(B)** Adsorption kinetics curves for Cr(VI) removal. Inset figure: pseudo-second-order fitting. Conditions: C0 = 400 ppm; sample volume: 10 ml; dosage: 1.0 g/L; pH 6.5; and temperature: 25°C. **(C)** Isotherms of HCSS on Hg (II) removal. **(D)** Isotherms of HCSS on Cr (VI) removal. Inset figure: Langmuir model fitting. Conditions: sample volume: 10 ml; dosage: 1.0 g/L; reaction time: 24 h; pH 6.5; and temperature: 25°C.

The adsorption kinetics of Hg (II) and Cr (VI) immobilization by HCSS was investigated, and the results are schemed in Figure 2. The reaction reached equilibrium approximately within 480 min for Hg (II), while the adsorption of Cr (VI) attained equilibrium within 360 min. The three-dimensional porous network structure on the surface of HCSS and multiple mechanisms are responsible for the adsorption of Hg (II) and Cr (VI) from the aqueous system (Jung and Sohn, 2014). Here, the pseudo-first-order and pseudo-second-order were employed to analyze the rate-determining step and adsorption mechanism of pollutants removal by HCSS. The corresponding parameters and correlation coefficient of each model are presented in Table 3. The plots of t/qt versus t exhibited excellent linearity in the pseudo-second-order model (Figure 2A inset). The correlation coefficients (R^2^) were calculated from equation 2 and equation 3, and the R^2^ of the pseudo-first-order model [0.870 and 0.882 for Hg (II) and Cr (VI), respectively] were lower than the pseudo-second-order model [0.996 and 0.993 for Hg (II) and Cr (VI), respectively]. Meanwhile, the calculated q_e_ in the pseudo-second-order model [276.25 and 323.155 mg/g for Hg (II) and Cr (VI), respectively] fitted well with the experimental data [283.24 and 320.12 mg/g for Hg (II) and Cr (VI), respectively]. Alternatively, the q_e_ values in the pseudo-first-order model [238.53 and 425.98 mg/g for Hg (II) and Cr (VI), respectively] were far from the experimental data. Hence, the kinetic results of Hg (II) and Cr (VI) removal highly suggested the pseudo-second-order model, which defined the adsorption of mercury and chromium on the HCSS was highly controlled by chemisorption. In literature, the rate-determining steps of chemical sorption involved chemical reduction, inner-sphere complexation, and coprecipitation. In this work, the experimental evidence of chemical reduction, inner-sphere complexation, and coprecipitation was also observed and will be discussed in the mechanism Section 3.4.

**TABLE 3:** Parameters of adsorption kinetics and isotherm models for Hg(II) and Cr(VI) removal by HCSS.

	Adsorption	Parameter	Heavy metal	
			Hg (Ⅱ)	Cr (Ⅵ)
Kinetics	Pseudo-first-order	k_1_ (min^−1^)	0.004	0.011
		Q_e_ (mg·g^−1^)	238.53	425.98
		R^2^	0.870	0.882
	Pseudo-second-order	k_2_ (mg·g^−1^·min^−1^)	0.003	0.003
		Q_e_ (mg·g^−1^)	276.25	323.155
		R^2^	0.996	0.993
Isotherms	Langmuir	k_L_ (L·mg^−1^)	0.012	0.009
		Q_m_ (mg·g^−1^)	281.31	322.18
		R^2^	0.997	0.998
		n	0.201	0.101
	Freundlich	K_F_ (mg·g^−1^)	32.17	41.10
		R^2^	0.871	0.863

In addition to the kinetics, adsorption mechanisms for Hg (II) and Cr (VI) removal were further explored *via* isotherm studies. As revealed, the adsorption capacities (Q_e_) increased along with the increasing of initial concentration (C_e_), and finally got hold of a plateau and achieved the Q_e_ of 283.24 and 320.12 mg/g for the adsorption of Hg (II) (Figure 2C) and Cr (VI) (Figure 2D), respectively. A total of two illustrative adsorption models, the Langmuir and Freundlich models, were conducted to pretend the reaction isotherm and reveal the nature of the adsorptive reactions as calculated by Eqs. (4, 5), respectively. Related parameters of Langmuir and Freundlich isotherm models were calculated and are listed in Table 3, while the Langmuir and Freundlich models were referred to the homogeneous monolayer and heterogeneous multilayer reactions of pollutants removal by sorbents, respectively (Rao et al., 2017). The value of Qe calculated by the Langmuir model was 281.31 and 322.18 mg/g for Hg (II) and Cr (VI), correspondingly, which was highly consistent with the experimental data [283.24 and 320.12 mg/g for Hg (II) and Cr (VI), respectively]. While the capacity calculated by the Freundlich model (K_F_) was 32.17 and 41.10 mg/g for Hg (II) and Cr (VI), respectively, and this K_F_ was far lower than that of experimental data. Meanwhile, the correlation coefficients (R^2^) calculated by the Langmuir model was 0.997 and 0.998 for Hg (II) and Cr (VI), respectively, which was higher than that of the Freundlich model [0.871 and 0.863 for Hg (II) and Cr (VI), respectively], implying the functional feasibility of the Langmuir model. These results demonstrated the homogeneous surfaces along with monolayer chemical reactions played a pivotal role in the elimination of heavy metals, leading to the superior adsorption capacity for the uptake of Hg (II) and Cr (VI) from aqueous solution.

To sum up, the Hg (II) and Cr (VI) removal by HCSS was mainly on homogeneous surfaces followed with the monolayer sorption. Moreover, after chemisorption of Hg (II) and Cr (VI) onto the surface of HCSS, the surface of sorbents were positively charged, if another heavy metal ion from solution was in contact with HCSS surface of positive charge, then repulsion was possible. Hence, it was not applicable for the fitting of multilayer adsorption in the Freundlich model.

#### 3.2.3 Influence of pH

**FIGURE 3:**
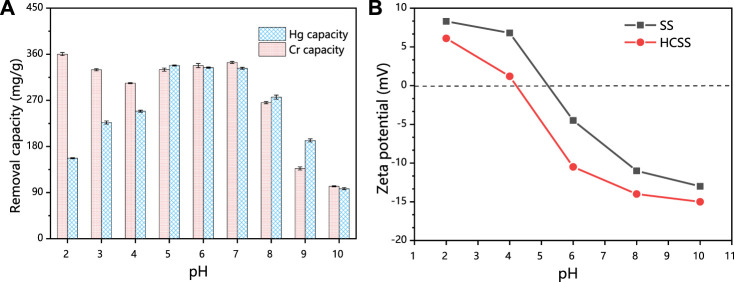
**(A)** Comparison of different pHs. Conditions: C0 = 400 ppm; sample volume: 10 ml; reaction time: 24 h; and temperature: 25°C. **(B)** Comparison of zeta potential of HSS and HCSS.

Initial solution pH affects the speciation of heavy metal ions, surface charge, and the ionization degree of the functional groups of the steel slag–derived materials, which in turn may strongly influence the adsorption performance, and also offer insights into the adsorption mechanisms. HCSS was employed for the elimination of Hg (II) and Cr (VI) from aqueous solution, and the determination of pH influence was conducted by varying the initial solution pH from 2 to 10. From Figure 3A, in the case of Hg (II), the capacity increased from 157.1 to 248.9 mg/g with the increase in pH from 2 to 4, and then capacity fluctuated between 338.1 and 332.6 mg/g at pH 5–6. Further increase in pH resulted in a decrease of the capacity from 276.4 mg/g (at pH 8) to 97.7 mg/g (at pH 10). However, as for Cr (VI), the capacity maintained with the increase in pH from 2 to 6 (360.3, 329.7, 303.6, 330.0, and 337.9 mg/g for pH 2, 3, 4, 5, and 6, respectively) and decreased from 344.1 to 102.3 mg/g with the further increase in pH from 7 to 10.

To explain the immobilization of Hg (II) and Cr (VI) by HCSS at various pHs, zeta potential was conducted (Figure 3B). The pH_ZPC_ (point of zero charges) of HCSS was determined to be 4.22, while that value of SS was 5.21, which further confirmed that the organic component was successfully assembled onto the surface of slag skeleton, due to the organic functional groups typically negatively charged (He et al., 2017). Moreover, in the case of Hg (II) adsorption, the surface of HCSS was positively charged at pH 2 (pH < pH_ZPC_); this positively charged surface of adsorbent created the electrostatic repulsion between the HCSS surface sites and cationic Hg (II), and the adsorption of Hg (II) was constrained, since Hg^2+^ is the only dominating species when pH < 4. Above this pH, Hg^2+^, Hg(OH)^+^, Hg(OH)_2_, and Hg(OH)_3_^−^ coexisted in the solution, the electronegative property of HCSS created the electrostatic attraction for mercury ions. Additionally, the functional groups were also deprotonated and formed complexation with mercury ions (such as COOHg^+^ or COHg^+^) (Gong et al., 2014), and all of these resulted in higher removal of Hg (II) ions at pH 5–6. However, when pH kept increasing, the adsorption capacity dropped dramatically, since the dominating species of Hg (II) ions were Hg(OH)^+^, Hg(OH)_2_, and Hg(OH)_3_^−^, and carriage of these species from solutions to the surface of adsorbents may be inhibited as the high molecular weight and large size of these species. Also, the loaded mercury species hindered the further adsorption between mercury ions with active sites on the surface of HCSS.

**FIGURE 4:**
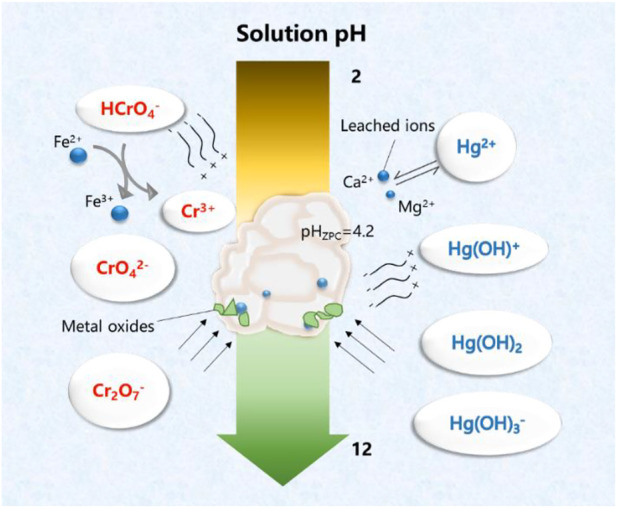
Conceptual representation of interactions between HCSS with mercury and chromium species in different solution pHs.

On the another hand, in the case of Cr (VI) removal, there was no noticeable decrease of capacity at pH 2–6, and this could be attributed to the dominant species of chromium ions were HCrO_4_^−^ and CrO_4_^2−^ in this pH environment; as they were negatively charged, they can create electrostatic attraction with HCSS. In addition, there were also more active species leaching from HCSS into solutions under acid conditions, such as calcium and iron, which was evidenced by the aforementioned leaching experiments. These active species could react with chromium ions and are beneficial to the removal of Cr (VI) (Xu et al., 2019), which would be discussed in the following sections. However, at pH > 7, the surface of sorbent was deprotonated and formed an electronegative potential. Thus strong electrostatic repulsion resulted from the electronegative HCSS surface and dominating chromium species, such as HCrO_4_^−^, Cr_2_O_7_^2−^, and CrO_4_^2−^, thus decreasing the capacity of the adsorbent. The pH dependence of Hg (II) and Cr (VI) adsorption corresponded to the surface charge of HCSS and the metal speciation in different pH environment (Figure 4). Therefore, neutral pH (6.5) was applied for further experiments for the Hg (II) and Cr (VI) removal.

### 3.3 Real water matrix

#### 3.3.1 Stability and reusability

**FIGURE 5:**
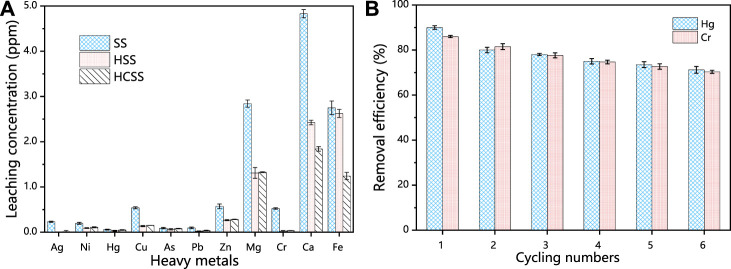
**(A)** Comparison of the different indigenous metal leaching concentration of SS, HSS, and HCSS. Conditions: sample volume: 10 ml; dosage: 100.0 g/L; reaction time: 48 h; pH 6.5; and temperature: 25°C. **(B)** Regeneration of HCSS.

For the safety concern, we also tested the secondary leaching performance of indigenous metallic from this slag-derived adsorbent. As shown in Figure 5A, for SS, the leaching amount was 3.342, 4.830, and 2.751 ppm for Mg, Ca, and Fe and 1.023, 0.571, and 0.540 ppm for Cr, Zn, and Cu, respectively. These values were similar to other reports (Han et al., 2016). However, after being converted into HSS *via* hydrothermal treatment, the leaching concentration of Mg, Ca, and Fe moderately decreased to 1.280, 2.402, and 2.616 ppm, correspondingly. This phenomenon could be attributed to the heavy metals were partially involved with the reconstruction processes of crystalline structure, as the increased number of network-forming metal oxides units under hydrothermal conditions (Dubey et al., 2017). Moreover, after being incorporated with biomass feedstock provided by SD, the resultant HCSS illustrated the limited leaching concentration as 0.190, 0.311, and 0.961 ppm for Mg, Ca, and Fe, respectively. This decrease in leaching was proposed to the chemical bonding between metals with functional groups on the surface of HCSS followed with the complete reconstruction of the crystalline structure, which was in a good agreement with the XRD analysis in the following sections. Compared with the original steel slag, the hydrochar component has a non-uniform surface, large specific surface area, numerous pores, rich organic functional groups, and the steel slag has a stable skeleton, inorganic adhesive for organic functional groups provide a rich active site, both firmly anchored in the process of heat and water together, and appear to have multiple interactions between the additional components, Therefore, HCSS has a stable structure and can inhibit metal leaching. Meanwhile, for the other metallic elements, such as Ag, Ni, Hg, Cu, As, Pb, Zn, and Cr, the leaching was only 0.023, 0.062, 0.001, 0.007, 0.024, 0.027, 0.023, and 0.015 ppm, respectively. These values were all lower than the recommended limitations by the World Health Organization (Ahmed and Ahmaruzzaman, 2016), implying the neglectable environmental negative impacts on water bodies.

A promising and competitive adsorbent should exhibit excellent recycling ability for practical applications. After six regeneration cycles, the removal efficiency was 71.2% for Hg (II) and 70.3% for Cr (VI), implying the excellent recoverable abilities for HCSS (Figure 5B). It should be mentioned that, in recent studies, the dominant mechanism for pollutants removal by slag-derived adsorbent was the leaching of chemical active species, such as leached Fe (II) for Cr (VI) reduction, or leached Ca (II) for phosphate precipitation (Fang et al., 2018). The main drawback was the lack of recyclability which limits the applications of these sorbents. However, in our study, this facile and green co-hydrothermal process successfully assembled hydrochar component onto the surface of slag skeleton; the hydrochar component was the main contributor to the capacity, and recrystallized steel slag as support, in turn, increased the stability and reusability of the adsorbent.

#### 3.3.2 Selectivity

**FIGURE 6:**
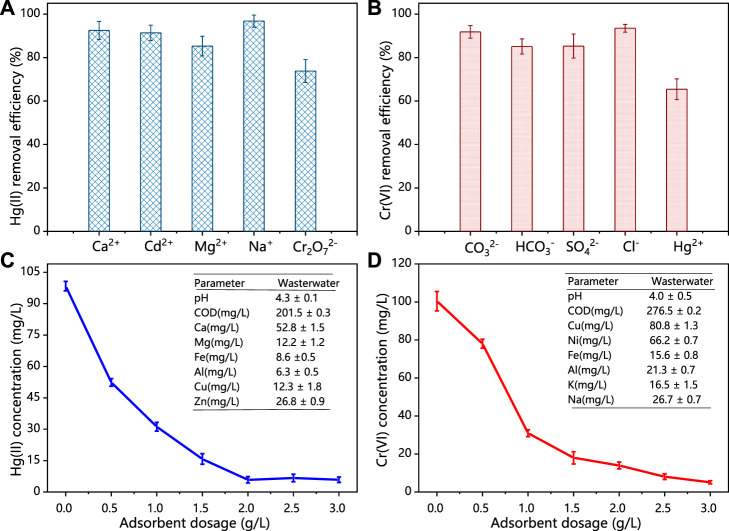
**(A)** Adsorption of Hg (II) by HCSS toward the mixture of metal ions. **(B)** Adsorption of Cr (VI) by HCSS toward the mixture of negatively charged ions. Conditions: concentration of competitive ions: 200 ppm; sample volume: 10 ml; dosage: 0.5 g/L; reaction time: 24 h; pH: 6.5; and temperature: 25°C. **(C)** Practical applications of HCSS for Hg (II) removal in real water. **(D)** Cr (VI) removal in real wastewater. Conditions: sample volume: 10 ml; reaction time: 24 h; and temperature: 25°C.

Coexisted metal ions in the practical wastewater system could influence the efficiency of adsorbents. Therefore, different metal ions, including Ca (II), Cd (II), Mg (II), Na (I), and K (I) from their chlorides, nitrates, and sulphates were added into solution, in order to evaluate the selective adsorption of HCSS for Hg (II) and Cr (VI) removal. As shown in Figure 6A, in the case of Hg (II) removal, the existence of competitive ions exhibited little influence for removal performance. The removal efficiency was 92.5%, 91.4%, 85.3%, 96.8%, and 73.8% for Ca^2+^, Cd^2+^, Mg^2+^, Na^+^, and Cr_2_O_7_^2−^, respectively, which demonstrated the good selectivity of HCSS on Hg (II) removal. It should be noticed that in the presence of Cr_2_O_7_^2−^, the removal efficiency on Hg (II) removal was still 73.8%, indicating the difference on reaction mechanism between Hg (II) and Cr (VI) removal. The decreasing removal efficiency for all coexisted metal ions might be attributed to the formation of some insoluble precipitates with sulfates or chlorides in the aqueous media (Lv et al., 2019). In addition, similar phenomenon was observed in Cr(VI) removal (Figure 6B), the removal efficiency was maintained as 91.8%, 85.1%, 85.3%, 93.5%, and 65.4% for CO_3_^2−^, SO_4_^2−^, Cl^−^, and Hg^2+^, respectively, indicating the good selectivity of HCSS for Cr (VI) removal among various negatively charged groups. The removal efficiency of Cr (VI) was reduced to 65.4% with the presence of Hg (II) might be attributed to the identical removal mechanisms, including formation of complexations and coprecipitation between Hg (II) ions with HCSS. However, the remaining Cr (VI) removal efficiency was contributed by the distinct mechanisms, such as chemical reduction, which would be discussed in the following section.

#### 3.3.3 Practical wastewater test

In order to further evaluate the practicability of HCSS in real water systems, the adsorbent was conducted to selectively capture Hg (II) or Cr (VI) from different practical wastewater. As shown in Figure 6 C, D, within the presence of excess competitive cations and anions, the concentration of Hg (II) was decreased from 98.4 to 5.9 mg/L with HCSS dosage increased from 0 to 3.0 g/L. As for Cr (VI), with the dosage of HCSS increased from 0 to 3.0 g/L, the Cr (VI) concentration decreased from 100.4 to 5.1 mg/L. These results further supported the Hg (II) or Cr (VI) removal by HCSS from real wastewater was less influenced by the variable chemistry and organic matter. Therefore, the obtained removal efficiency as 94.11% for Hg (II) and 88.65% for Cr (VI) elimination from practical wastewater, indicated the excellent feasibility and applicability of HCSS on real wastewater treatment.

#### 3.3.4 Comparison study on the adsorption capacity

Various environmentally functional materials were compared on the adsorption capacity and reaction parameters for the removal of Hg (II) and Cr (VI) and summarized in Table 4. HCSS displayed superior adsorption capacity of 283.24 mg/g for Hg (II) with a dosage of 1.0 g/L, while the capacity of 320.12 mg/g for Cr (VI) with a dosage of 1.0 g/L. The high efficiency for Hg (II) and Cr (VI) removal implied this simple one-pot method was an emerging and promising method for modification of solid waste-derived materials.

**TABLE 4:** Comparison of adsorption capacities of Hg(II) and Cr(VI) ions with some other functional materials.

Raw material	Modification	Condition		Maximum capacity (mg/g)		Equilibrium time	Reference
		Temperature (°C)	pH	Hg(II)	Cr(VI)		
Chitosan	Ethylhexadecyldimethyl ammonium bromide-impregnated chitosan	25	3.0	43.43	NA	5 h	Shekhawat et al. (2017)
Sawdust	Hydrothermally treated magnetic bio-adsorbents	25	6.5	167.2	NA	6 h	Wang et al. (2018)
Sawdust	Monomethylated thiourea sawdust	30	6	4.26	NA	4 h	Hashem et al. (2011)
Sewage sludge	Carboxymethyl chitosan–sewage sludge biochar	30	3	594.17	NA	4 h	Ifthikar et al. (2018)
Montmorillonite	Chitosan–nanoclay composite	30	2	NA	128.43	2 h	Kahraman, (2017)
Montmorillonite	Interaction of surfactant–modified sodium montmorillonite	30	3.8	NA	22.2^a^	80 min	Kumar et al. (2012)
Biomass	Nitric acid–activated carbon	25	6	NA	48	24 h	Valentín-Reyes et al. (2019)
Biomass	Chitosan modification of magnetic biochar	30	2	NA	120	200 min	Zhang et al. (2015)
Furnace slag	Ground and sieved, and H_2_SO_4_ was added into the solution to form CaSO_4_·H_2_O	25	3	NA	12.3	70 min	Han et al. (2016)
Furnace slag	NaOH-activated slag	25	3	NA	6.7	4 h	Baalamurugan et al. (2018)
Steel slag	Incorporation of sawdust with steel slag by co-hydrothermal process	25	6.5	283.24	320.12	6 h for Hg (II)	This study
						5 h for Cr (VI)	

NA: not applicable.

a Calculated maximum capacity.

### 3.4 Adsorption mechanisms

#### 3.4.1 Recrystallization

**FIGURE 7:**
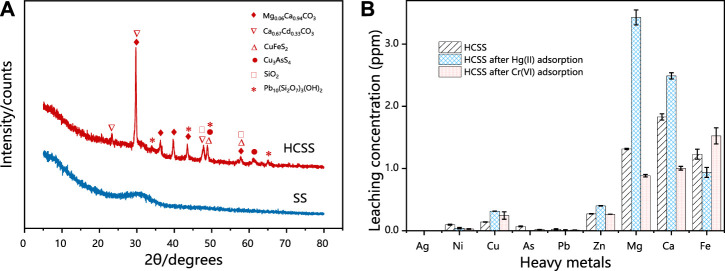
**(A)** XRD patterns of SS and HCSS. **(B)** Comparison of the different metal leaching concentrations of HCSS after Hg (II) and Cr (IV) removal. Conditions: C0 = 400 ppm; sample volume: 10 ml; dosage: 100.0 g/L; reaction time: 48 h; pH: 6.5; and temperature: 25°C.

The reconstruction of inorganic slag skeleton was confirmed by XRD (Figure 7A). In the case of SS, only a broad peak was observed in the range of 25.0°–35.0°, which might be attributed to the irregular structure of various indigenous metal oxides in SS. Moreover, after co-hydrothermal with biomass feedstock, the sharp and robust reflection peaks were observed for HCSS, peaks at 2ϴ of 29.7°, 36.5°, 39.7°, 43.7°, and 57.6° indexed to Mg_0.06_Ca_0.94_CO_3_ (JCPDS No. 01-089-1306)while peaks at 47.9° and 57.7° were ascribed to the formation of SiO_2_ (JCPDS No. 00-047-1301). The alteration of characteristic reflection peaks between SS and HCSS proved the completion of the reconstruction process. The functional groups provided by biomass feedstock exhibited excellent affinity with surface metal ions, and the existence of CO_3_^2−^ in the Ca_0.67_Cd_0.33_CO_3_ and Mg_0.06_Ca_0.94_CO_3_ oxides might be attributed to the organic functional groups involved with recrystallization process (Santamaria et al., 2018). Moreover, the reformed crystalline structure was also helpful to the adsorptive reactions for Hg (II) and Cr (VI) removal, due to the coprecipitation process between pollutants with metal oxides (Richard et al., 2016). Additionally, some crystalline structures observed in HCSS, such as Ca_0.67_Cd_0.33_CO_3_ (JCPDS No. 01-072-1938), Cu_3_AsS_4_ (JCPDS No. 00-025-0265), and Pb_10_ (Si_2_O_7_) _3_ (OH) _2_ (JCPDS No. 00-044-0276), could reduce the potential leaching of toxic metal ions, which in turn, increased the safety and stability of HCSS (Liu et al., 2016). This result was also in good agreement with the previous indigenous metal leaching test.

Surface morphology was characterized by SEM-EDX (Supplementary Figure S3). HSS was smoother with fewer pores than SS, while SS exhibited chiseled microspore structure. This stable crystalline structure of HSS could be potential support for the assembly of surface functional groups. In addition, according to EDX results, HSS exhibited higher contents of Ca (31.63 wt%) and Fe (2.31 wt%) than SS (24.44 wt% and 0.23 wt% for Ca and Fe, respectively), this could be explained as the recrystallization benefited the exposure of indigenous metallic elements encapsulated in the lattice (Ma et al., 2018). As mentioned before, the high ratio of metallic ions on the surface of support might be beneficial to the subsequent assembly or reaction process.

The distribution of the surface metallic elements was further detected *via* X-ray fluorescence (Supplementary Figure S4). It is mainly composed of CaO, SiO_2_, and Al_2_O_3_ on the surface of HCSS, with the content of 36.85, 39.28, and 14.58 wt%, correspondingly. The existence of calcium ions on the surface of HCSS could be involved in ion-exchange with Hg (II) ions during the adsorption process. The formation of inner-sphere complexation between silica and heavy metals was also beneficial to the adsorption process. More importantly, the iron ions on the surface of HCSS existed as Fe (II) species, and the content of FeO was 2.92 wt%, these species might exhibit reductive property for Cr (VI) immobilization (Hu et al., 2019a).

#### 3.4.2 Surface functional groups

**FIGURE 8:**
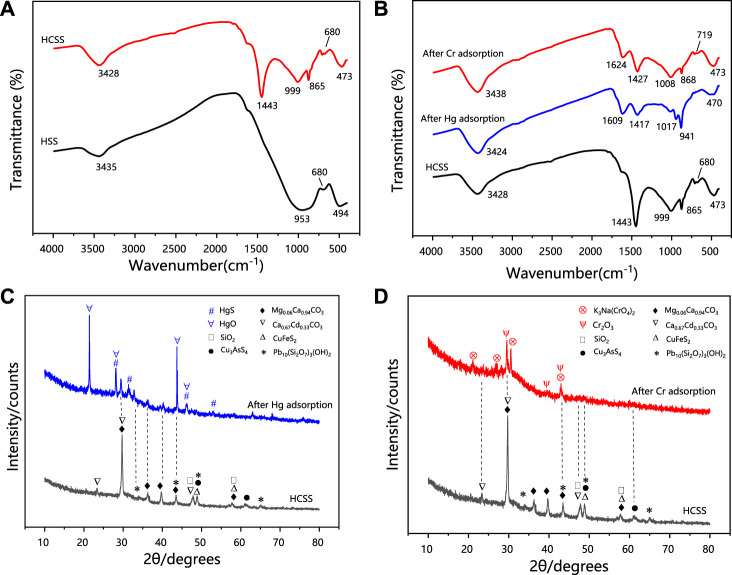
**(A)** FT-IR patterns of HCSS before adsorption. **(B)** FT-IR patterns of Hg-adsorbed HCSS, Cr-adsorbed HCSS, and comparison with HCSS. **(C)** XRD patterns of Hg-adsorbed HCSS. **(D)** XRD patterns of Cr-adsorbed HCSS.

To further investigate the variations of surface functional groups, FT-IR spectra were illustrated in Figure 8A. The signal at 3,435 cm^−1^ of HSS and 3,428 cm^−1^ of HCSS was attributed to the vibration of O-H (Rao et al., 2017), which evidenced the existence of -OH groups. The band at 1,443 cm^−1^ of HCSS can be endorsed to stretching of N-H, and 865 cm^−1^ was associated with the vibration of C = O (Li et al., 2015), which were both ascribed to the functional groups from the incorporation of bioresource, and were only observed on the surface of HCSS. In the case of HSS, the signal at 953 cm^−1^ was attributed to the vibration of Si-O groups, and this signal was shifted into 999 cm^−1^ in the case of HCSS, indicating the Si-O structure was transformed, and new silica-based crystals might be generated (Pala et al., 2014). This reforming of Si-O contained structure during adsorbents preparation was further confirmed by XRD spectra in the following section. The peak at 680 cm^−1^ was ascribed to the bending vibration of Fe-O, implying the existence of iron on the surface both for HCSS and HSS (Tan et al., 2015). These outcomes of FT-IR spectra confirmed the successful loading of organic components onto the surface of the supporting slag skeleton, and in turn, as-prepared HCSS comprised carbon skeleton with a high ratio of hydroxyl, carboxyl, amino, and lactone functional groups, which was consistent with S_BET_ and Boehm analysis.

The FT-IR spectra of HCSS after adsorption of Hg (II) and Cr (VI) were presented in Figure 8B. After the adsorption, significant shifts of characteristics bands from 3,428 (stretching vibrations of O-H/N-H) to 3,424 and 3,438 cm^−1^, from 1,443 (bending vibration of N-H) to 1,417 and 1,427 cm^−1^, from 999 (bending vibration of Si-O) to 1,017 and 1,008 cm^−1^, from 865 (bending vibration of C = O) to 941 and 868 cm^−1^ was observed after the adsorption of Hg (II) and Cr (VI), respectively. These results illustrated the formation of surface complexations between pollutants and functional groups, and high functional groups density on the surface of HCSS improved the removal capacity, which further evidenced the functional groups on the surface was the main contributor to the removal capacity. However, the abundant functional groups only could be achieved by the co-hydrothermal process between biomass feedstock and inorganic skeleton, owing to the obtained regular backbone benefited to the adherence of functional groups. It should be noticed that the peak at 680 cm^−1^ in HCSS was ascribed to the bending vibration of Fe-O. After Cr (VI) adsorption, this peak shifted to 719 cm^−1^, and this transformation was not observed in the case of Hg (II) removal (Wang et al., 2018). This phenomenon further proved the chemical reduction between iron and chromium ions, as the dominant iron oxides might be converted from FeO into Fe_2_O_3_ after Cr (VI) adsorption, which was also consistent with XRF results (Supplementary Figure S5).

#### 3.4.3 Electron transfer

XRD spectra further provided the evidence for the chemical interaction during the adsorption process. As revealed in Figures 8C,D, characteristic XRD patterns of HgS and HgO were observed for Hg (II) removal with the typical 2ϴ of 21.2°, 28.2°, 43.7°, 46.1°, and 28.2°, 28.2°, 43.7°, 46.1°, respectively (JCPDS No. 01-073-1247). The reflection peaks at 2ϴ of 29.6°, 39.6°, and 43.0° indicated the existence of Cr_2_O_3_ in the case of Cr(VI) removal (JCPDS No. 01-081-1136). The formation of HgS, HgO, and Cr_2_O_3_ could be ascribed to the reactions between Hg (II), Cr (VI) with the metal oxides, while more regular crystalline oxide matrix could enhance the coprecipitation process (Deng et al., 2017). This reconstructed structure was obtained under the co-hydrothermal circumstance and considerably improved the crystallinity of the support. Moreover, the chromium species existed as Cr (III) in the resultant Cr_2_O_3_ demonstrated Cr (VI) could be reduced into Cr (III) by active chemical species (such as iron ions) during the adsorption (Hu et al., 2019). As illustrated in Supplementary Figure S5 XRF analysis of HCSS after the adsorption confirmed the existence of HgO (7.27 wt%), which was consistent with XRD results. More importantly, before adsorption, iron species existed as FeO in HCSS, however, after the adsorption of Cr (VI), the existence of Cr_2_O_3_ (7.32 wt%) and Fe_2_O_3_ (0.67 wt%) were confirmed, which further evidenced the reduction from Cr (VI) to Cr (III) by Fe (II) species during the adsorptive reactions.

Furthermore, redox potential, E^0^, a crucial measure of the tendency of a chemical species to acquire from or lose electrons further reveal the mechanism of Cr (VI) reduction process. Herein, E^0^ of Cr_2_O_7_^2−^/Cr^3+^, O_2_/OH^−^, and Fe_3_O_4_/Fe_2_O_3,_ was 1.33, 0.41 and 0.215 V, respectively (Ciblak et al., 2012), indicating Cr_2_O_7_^2−^/Cr^3+^ could easier react with FeO (Fe_3_O_4_) compared with oxidized by oxygen. Therefore, the reduction process could be explained by the following equations:

$$\begin{array}{c} 6\mathrm{Fe}O+{\mathrm{Cr}}_{2}O_{7}^{2-}+8H^{+}\to 2{\mathrm{Cr}}^{3+}+3{\mathrm{Fe}}_{2}O_{3}+4{\text{H}}_{2}O, \end{array}$$ (6)

$$\begin{array}{c} 2\left(2\mathrm{Ca}\cdot {\mathrm{SiO}}_{2}\right)+4{\text{H}}_{2}O\to 3\mathrm{Ca}\cdot 2{\mathrm{SiO}}_{2}\cdot 3{\text{H}}_{2}O+\mathrm{Ca}{\left(\mathrm{OH}\right)}_{2}, \end{array}$$ (7)

$$\begin{array}{c} 3\left(3\mathrm{Ca}\cdot {\mathrm{SiO}}_{2}\right)+6{\text{H}}_{2}O\to 3\mathrm{Ca}\cdot 2{\mathrm{SiO}}_{2}\cdot 3{\text{H}}_{2}O+3\mathrm{Ca}{\left(\mathrm{OH}\right)}_{2}, \end{array}$$ (8)

$$\begin{array}{c} 2{\mathrm{Cr}}^{3+}+3\mathrm{Ca}{\left(\mathrm{OH}\right)}_{2}\to 2\mathrm{Cr}{\left(\mathrm{OH}\right)}_{3}+3{\mathrm{Ca}}^{2+}, \end{array}$$ (9)

$$\begin{array}{c} 2\mathrm{Cr}{\left(\mathrm{OH}\right)}_{3}\to \text{C}{\text{r}}_{2}O_{3}+3{\text{H}}_{2}O \end{array}.$$ (10)

#### 3.4.4 Multiple interactions

Based on the former analysis, both chemical and physical adsorption could contribute to the adsorption of Hg (II) and Cr (VI) onto HCSS. Based on the kinetics study, the initial fast stage was ascribed to the ion-exchange, electrostatic attraction, and chemical reduction, while the chemical process is the dominant step for rate determination (Sumaraj and Padhye, 2017). The chemisorption on the homogeneous surface along with monolayer reactions was also demonstrated by isothermal analysis, and more solid experimental evidence for the detailed chemical process might help to understand the superior adsorption capacity.

As evidenced by S_BET_ and SEM, the high surface areas and regular crystalline structure of HCSS enable this sorbent to be easily accessible to heavy metal ions and contribute to the further reaction (Takaya et al., 2016). The pH experiment and the measurement of pHZPC illustrated that the Hg (II) and Cr (VI) [reduced as Cr (III)] could be electrostatic attracted onto HCSS, due to the negatively charged surface of HCSS at pH 6.5 (Burton et al., 2019). Moreover, the more negatively charged property also confirmed the assembly of functional groups on the surface of the slag skeleton. The quantitative study on the concentration of metallic elements after Hg (II) and Cr (VI) adsorption was also conducted (Figure 7B). In the case of Hg (II) removal, the concentration of Mg (II) and Ca (II) after adsorption increased to 3.45 and 2.51 ppm, respectively. However, there was no noticeable increase of metal ions concentration after Cr (VI) adsorption [only 0.19 and 0.31 ppm for Mg (II) and Ca (II), respectively], which was attributed to the different charge density between chromium ions and other surface metal ions (Jobby et al., 2018). These verdicts illustrated that Hg (II) ions were adsorbed on HCSS by the process of ion-exchange through reaction Eqs. 11, 12 (Lu et al., 2012).

$$\begin{array}{c} -{\mathrm{Mg}}^{2+}+{\mathrm{Hg}}^{2+}\to -{\mathrm{Hg}}^{2+}+{\mathrm{Mg}}^{2+}, \end{array}$$ (11)

$$\begin{array}{c} -{\mathrm{Ca}}^{2+}+{\mathrm{Hg}}^{2+}\to -{\mathrm{Hg}}^{2+}+{\mathrm{Ca}}^{2+}. \end{array}$$ (12)

**FIGURE 9:**
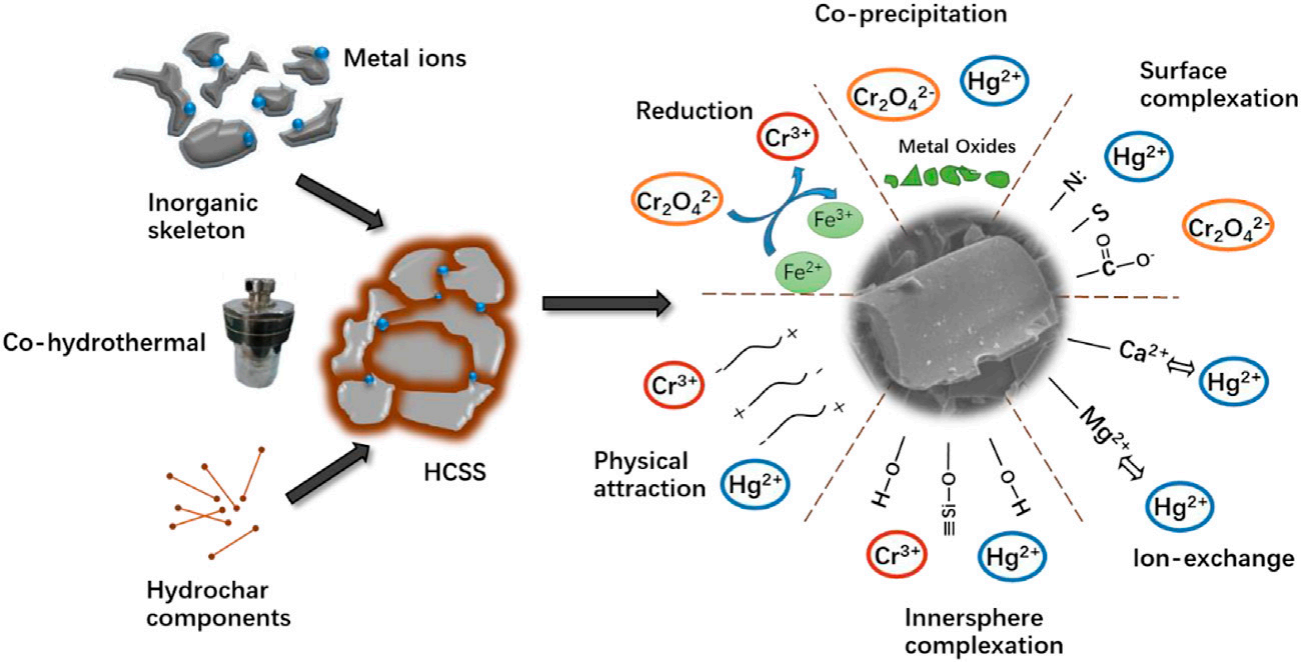
Conceptual sketch of Hg (II) or Cr (VI) removal by HCSS.

To sum up, the removal mechanisms for Hg (II) and Cr (VI) were proposed and demonstrated in Figure 9. After being simultaneously recrystallized and bioconverted *via* co-hydrothermal treatment, the biomass and slag exhibited a synergetic effect in Hg (II) and Cr (VI) removal. The adsorption process was a combination of physical attractions, the formation of inner-sphere complexations with hydroxyl, exchanges with internal cations, the formation of surface complexations with functional groups, coprecipitations, and formation of Si-O complexations. All these mechanisms contributed to the efficient performance of Hg (II) and Cr (VI) removal.

## 4 Conclusion

1) Among all sorbents (SS, HSS, HSD, HCSS, and physical mixture of HSS/HSD and SS/HSD) investigated, HCSS exhibited the superior capacity for Hg^2+^ and Cr_2_O_7_^2−^ removal. This environmentally functional material illustrated an exceptional capacity (283.24 and 323.16 mg/g for Hg (II) and Cr (VI) removal, respectively) compared with other adsorptive materials.
2) The high capacity mainly originates from converted hydrochar components, but the excellent stability and reusability of HCSS were attributed to the physiochemically stable inorganic skeleton derived from steel slag, which was also recrystallized under the hydrothermal circumstance.
3) Cr (VI) could be chemically reduced by the indigenous reactive species in slags, such as Fe (II). The adsorption was pH-dependent, owing to the multiple mechanisms, such as rate-determining steps of chemisorption including chemical reduction, coprecipitation, inner-sphere complexation, and involved with other processes, such as ion-exchange and electrostatic attraction.
4) HCSS exhibited good selectivity on heavy metals removal, and this material was also proved satisfied with removal efficiency for real wastewater treatment, with the percentage of 94.11% and 88.65% for Hg (II) and Cr (VI), respectively, indicating the potential practical feasibility of this environmentally functional materials.
5) This simultaneous recrystallization and bioconversion process shed the new light for the support design for carbonaceous materials and reutilization of steel slag, and this co-hydrothermal treatment could be considered an emerging and promising approach for the synthesis of other solid waste-derived environmentally functional materials.

## Data Availability

The original contributions presented in the study are included in the article/Supplementary Materials; further inquiries can be directed to the corresponding authors.
