# Supplementary material for: Efficient removal of mercury and chromium from wastewater via biochar fabricated with steel slag: Performance and mechanisms
Source: Front Bioeng Biotechnol. 2022 Aug 25;10:961907. doi: 10.3389/fbioe.2022.961907 (PMC9453161; doi:10.3389/fbioe.2022.961907)
Supplement: Supplementary file 1 [file Datasheet1.docx]

**Supplementary material**

**Efficient removal of mercury and chromium from wastewater via biochar fabricated with steel slag: Performance and mechanisms**

***Huabin Wang^1,2^, Ran Duan^1^, Xinquan Zhou^2, 3^, Jia Wang^2^, Ying Liu^1^,*** ***Rui Xu^1*^, Zhuwei Liao^2,4*^***

*^1^School of Energy and Environment Science, Yunnan Normal University, Kunming, China, ^2^Department of Environmental Engineering, School of Environmental Science and Engineering, Huazhong University of Science and Technology, Wuhan, China, ^3^School of Chemical Engineer and Pharmacy, Henan University of Science and Technology, Luoyang, China; ^4^Urban Construction Engineering Division, Wenhua College, Wuhan, China*

*** Correspondence:**Rui Xu
ecowatch_xr@163.com

Keywords: Biochar, Steel slag, Mercury, Chromium, Adsorption.

**Contents**

**Fig. S1.** Photographs of supernatants after adsorption of Hg(II).

**Fig. S2. (A**) Influence of different preparation ratio **(B)** Influence of different heat treatment temperature for removal capacities.

**Fig. S3. (A)** SEM image of SS **(B)** EDX scanning results of SS **(C)** SEM image of HCSS **(B)** EDX scanning results of HCSS.

**Fig. S4.** XRF results of HCSS before the adsorption.

**Fig. S5. (A**) XRF results of HCSS after Hg adsorption **(B)** XRF results of HCSS after Cr adsorption.

**
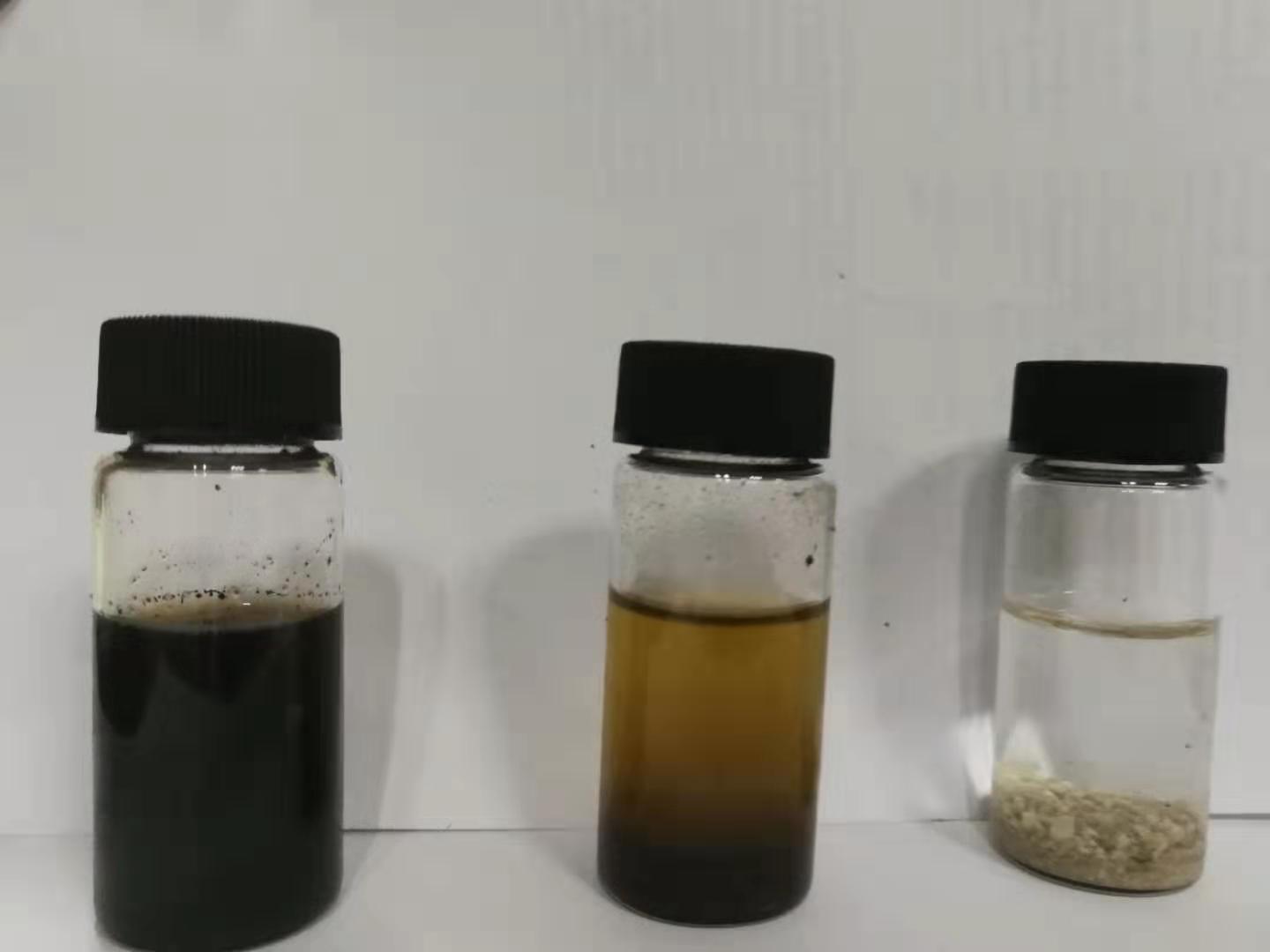
**

HSS

HCSS

HSD

**Fig. S1.** Photographs of supernatants after adsorption of Hg(II).

**Fig. S2. (A**) Influence of different preparation ratio. **(B)** Influence of different heat treatment temperature for removal capacities. Conditions: sample volume 10 mL, dosage 1.0 g/L, C_0_ = 100 ppm, reaction time = 24 h, pH 6.5 and temperature 25 ˚C.

**
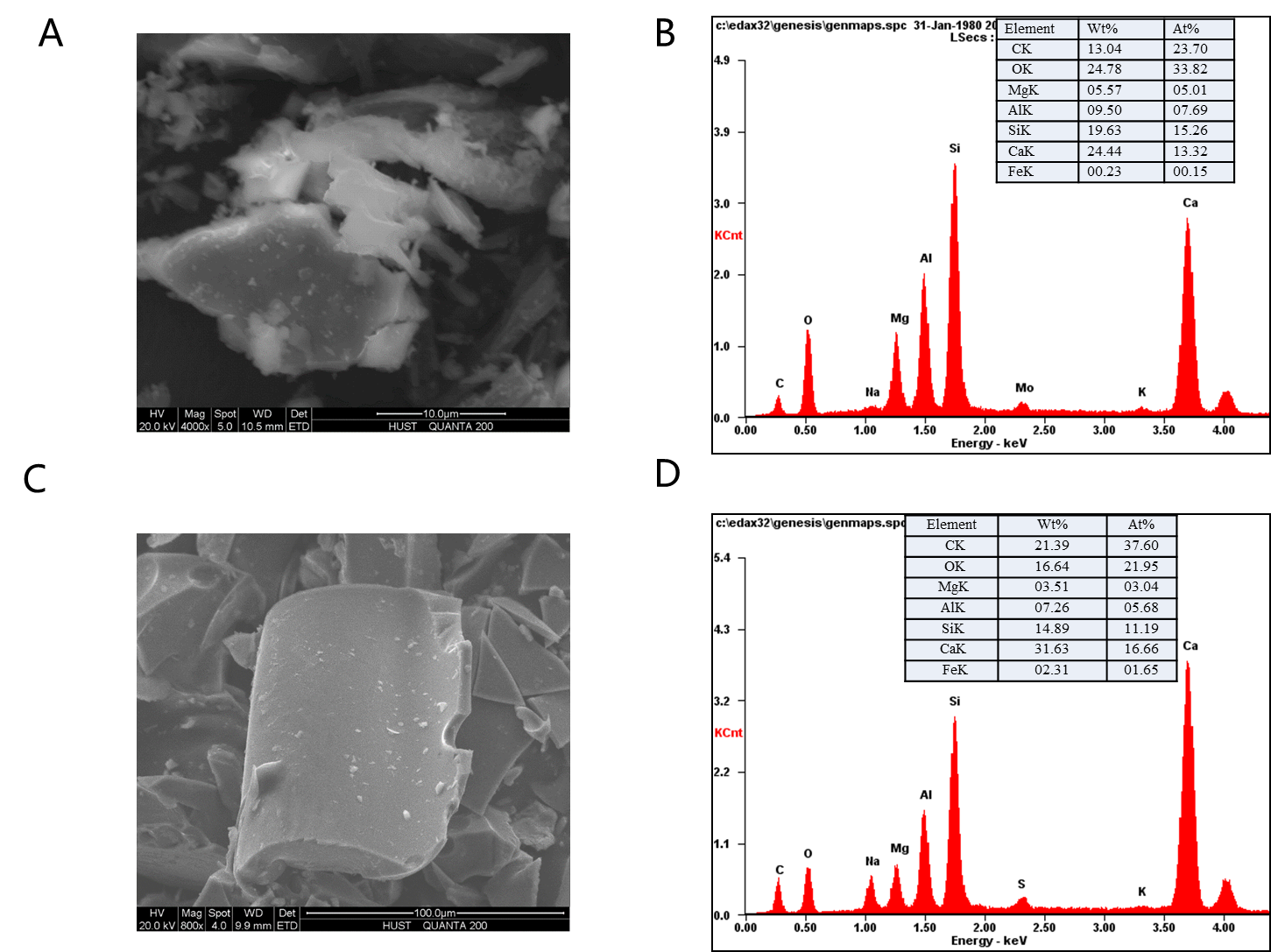
**

**Fig. S3 (A)** SEM image of SS **(B)** EDX scanning results of SS **(C)** SEM image of HSS **(B)** EDX scanning results of HSS.

**
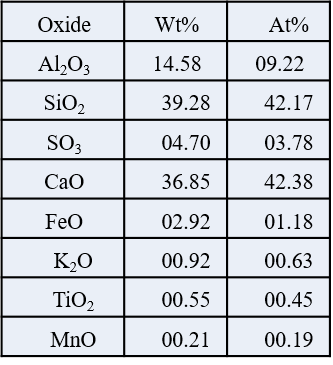

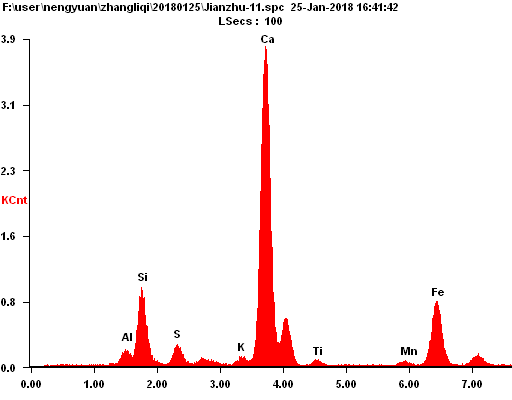
**

**Fig. S4.** XRF results of HCSS before the adsorption.

**
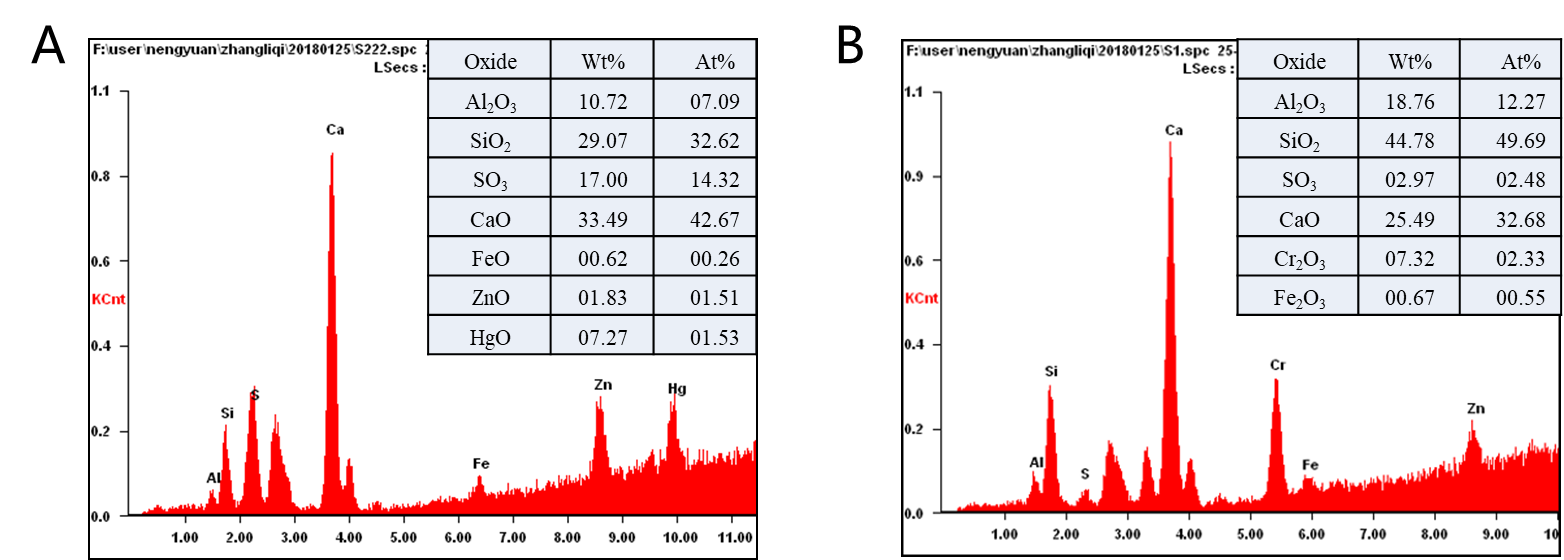
**

**Fig. S5. (A**) XRF results of HCSS after Hg adsorption **(B)** XRF results of HCSS after Cr adsorption.
